# Hyperthyroidism, acromegaly, and hyperprolactinemia in a patient with a mature plurihormonal PIT-1 lineage adenoma

**DOI:** 10.1210/jcemcr/luag129

**Published:** 2026-06-05

**Authors:** Julia Ferreira de Carvalho, Daniel Slack, Raj Shrivastava, Timothy E Richardson, Raquel Teixeira Yokoda, Alice C Levine

**Affiliations:** Division of Endocrinology, Diabetes and Bone Disease, Department of Medicine, Icahn School of Medicine at Mount Sinai, New York, NY 10029, USA; Division of Endocrinology, Diabetes and Bone Disease, Department of Medicine, Icahn School of Medicine at Mount Sinai, New York, NY 10029, USA; Department of Neurosurgery, Icahn School of Medicine at Mount Sinai, New York, NY 10029, USA; Department of Pathology, Molecular and Cell-Based Medicine, Icahn School of Medicine at Mount Sinai, New York, NY 10029, USA; Friedman Brain Institute, Icahn School of Medicine at Mount Sinai, New York, NY 10029, USA; Department of Pathology, Molecular and Cell-Based Medicine, Icahn School of Medicine at Mount Sinai, New York, NY 10029, USA; Department of Laboratory Medicine and Pathology, Mayo Clinic Arizona, Phoenix, AZ 85054, USA; Department of Neurological Surgery, Mayo Clinic Arizona, Phoenix, AZ 85054, USA; Division of Endocrinology, Diabetes and Bone Disease, Department of Medicine, Icahn School of Medicine at Mount Sinai, New York, NY 10029, USA

**Keywords:** plurihormonal pituitary adenoma, TSH-secreting adenoma, growth hormone-secreting adenoma, central hyperthyroidism, acromegaly

## Abstract

Concurrent thyrotropin (TSH) secreting and growth hormone (GH) secreting pituitary adenomas are rare entities. Although tumors of the PIT-1 lineage may coexpress TSH and GH, concurrent biologically active secretion resulting in central hyperthyroidism and acromegaly is uncommon. We report the case of a 42-year-old woman presenting with weight loss, palpitations, amenorrhea, and progressive acromegalic features. Biochemical evaluation revealed central hyperthyroidism with elevated free T4 and inappropriately normal TSH, markedly elevated insulin-like growth factor 1 (IGF-1) and GH, hyperprolactinemia, and elevated α-subunit. Pituitary magnetic resonance imaging demonstrated a 2.7-cm macroadenoma with cavernous sinus extension. She underwent transsphenoidal resection without complications. Pathology confirmed a mature plurihormonal PIT-1 lineage adenoma immunopositive for TSH, GH, and prolactin with a Ki-67 of 3%. Immediately postoperatively, thyrotoxicosis and hyperprolactinemia resolved, IGF-1 levels declined, acromegalic features improved, and menses resumed. Over the ensuing months, thyroid and prolactin tests remained within normal range, but the IGF-1 level remained elevated. This case illustrates the rare presentation of a PIT-1 lineage adenoma secreting biologically active TSH, GH, and prolactin. Comprehensive hormonal evaluation and long-term biochemical and radiologic surveillance are essential, given the risk of incomplete remission despite apparent surgical cure.

## Introduction

Thyrotropin (TSH) and growth hormone (GH) secreting pituitary adenomas are both rare entities, with an incidence of 1 per million people and 1 to 4 per million people, respectively [1, 2]. Pituitary adenomas exhibit plurihormonality, secretion of more than 1 pituitary hormone, in only a minority of cases [3]. The secretory capacity of a pituitary adenoma is generally determined by immunohistochemical assessment of tissue sections and is not always associated with increased serum hormone levels or clinical findings. Thyrotroph, somatotroph, and lactotroph cells have the same progenitor, regulated by the PIT-1 transcription factor [4]. Thus, it is not unusual for a TSH-, GH-, or prolactin-secreting pituitary adenoma to exhibit plurihormonality with other PIT-1–regulated hormones, as evidenced by immunohistochemical hormone expression. It is extremely rare, however, for these tumors to secrete multiple biologically active hormones with apparent clinical sequelae. Herein, we report on a case of a patient with a mature plurihormonal PIT-1 lineage pituitary adenoma secreting TSH, resulting in central hyperthyroidism, hyperprolactinemia, and concomitant production of GH, resulting in acromegaly.

## Case presentation

A 42-year-old woman with a 1-year history of amenorrhea presented to urgent care with 3 months of progressive weight loss, fatigue, palpitations, and tremulousness. Laboratory testing was consistent with central hyperthyroidism, with elevated free thyroxine (T4) (> 7.77 ng/dL [International System of Units (SI): 100 pmol/L] (reference range, 0.82-1.77 ng/dL [SI: 10.6-22.8 pmol/L]) and inappropriately normal TSH (3.25 mIU/mL) (reference range, 0.45-4.5 mIU/L). The patient was referred to endocrinology, and on initial evaluation, she noted a 3-month history of progressive jaw prominence, bilateral enlargement of hands and feet, and galactorrhea. The patient had a history of gestational diabetes and 1 prior pregnancy conceived by in vitro fertilization 8 months prior to presentation, during which she was unable to breastfeed. Her physical exam was notable for an enlarged tongue, jaw, hands, and feet; the presence of a goiter; intact extraocular movements without lid lag; and tachycardia with regular rhythm.

## Diagnostic assessment

A full pituitary panel was obtained and was again notable for central hyperthyroidism with elevated free T4 (5.02 ng/dL [SI: 64.6 pmol/L]) and TSH (4.68 mIU/mL). Prolactin was also elevated (122 ng/mL [SI: 2586.4 mIU/L]) (reference range, 4.8-23.3 ng/mL [SI: 101.8-494.0 mIU/L]), along with insulin-like growth factor 1 (IGF-1) (795 ng/mL [SI: 103.4 nmol/L]) (reference range, 91-308 ng/mL [SI: 11.8-40 nmol/L]), GH (19 ng/mL [SI: 19 µg/L]) (reference range, 0-10 ng/mL [SI: 0-10 µg/L]), and free α-subunit (28 ng/mL [SI: 28 µg/L] (reference range, <1.02 ng/mL [SI < 1.02 µg/L]). The α-subunit/TSH molar ratio was markedly elevated (approximately 60). Additionally, luteinizing hormone (LH) was 1.5 mIU/mL (SI: 1.5 IU/L) (reference range, 2.4-12.6 mIU/mL [SI: 2.4-12.6 IU/L]), follicle stimulating hormone (FSH) 4.1 mIU/mL (SI: 4.1 IU/L) (reference range, 3.5-12.5 mIU/mL [SI: 3.5-12.5 IU/L]). ACTH and cortisol were collected at noon and considered borderline: corticotropin (ACTH) was 46.4 pg/mL (SI: 10.2 pmol/L) (reference range, 7.2-63.3 pg/mL [SI: 1.6-13.9 pmol/L]), and cortisol was 4.2 µg/dL (SI: 115.9 nmol/L) (normal morning range, 6.7-22.6 µg/dL [SI: 184.9-623.7 nmol/L]). Albumin was 4.1 g/dL (SI: 41 g/L) (reference range, 3.8-4.8 g/dL [SI: 38-48 g/L]), and calcium was 9.8 mg/dL (SI: 2.45 mmol/L) (reference range, 8.7-10.2 mg/dL [SI: 2.17-2.55 mmol/L]). The patient underwent a thyroid ultrasound, which revealed a multinodular goiter with small, scattered nodules. The patient was started on atenolol 25 mg once daily to reduce the perioperative risk associated with uncontrolled hyperthyroidism.

**Figure 1:**
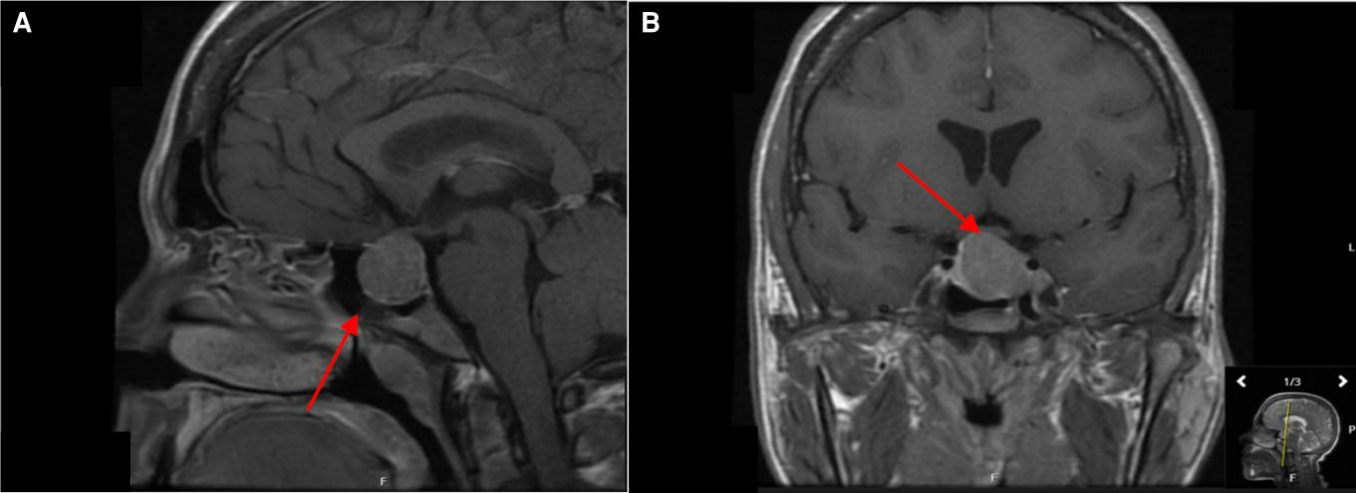
Postcontrast T1 magnetic resonance imaging demonstrating a homogenously enhancing sellar mass measuring 2.2 × 2.7 × 2.0 cm in the midsagittal (A) and coronal (B) planes.

A contrast-enhanced magnetic resonance imaging (MRI) of the pituitary was notable for a pituitary macroadenoma measuring 2.2 cm × 2.7 cm × 2.0 cm with asymmetric extension into the left cavernous sinus and mild elevation of the adjacent optic chiasm (Fig. 1).

## Treatment

The patient underwent an uncomplicated transsphenoidal resection of the tumor with postoperative MRI demonstrating no definite evidence of residual tumor. She remained hospitalized for 2 days after her surgery and did not develop arginine vasopressin deficiency or syndrome of inappropriate antidiuretic hormone. Given borderline preoperative cortisol and ACTH levels, she was discharged on oral hydrocortisone 10 mg each morning and 5 mg each afternoon. On postoperative day 1, TSH decreased to 0.095 µIU/mL; therefore, the patient was discharged on oral levothyroxine 50 mcg daily.

**Figure 2:**
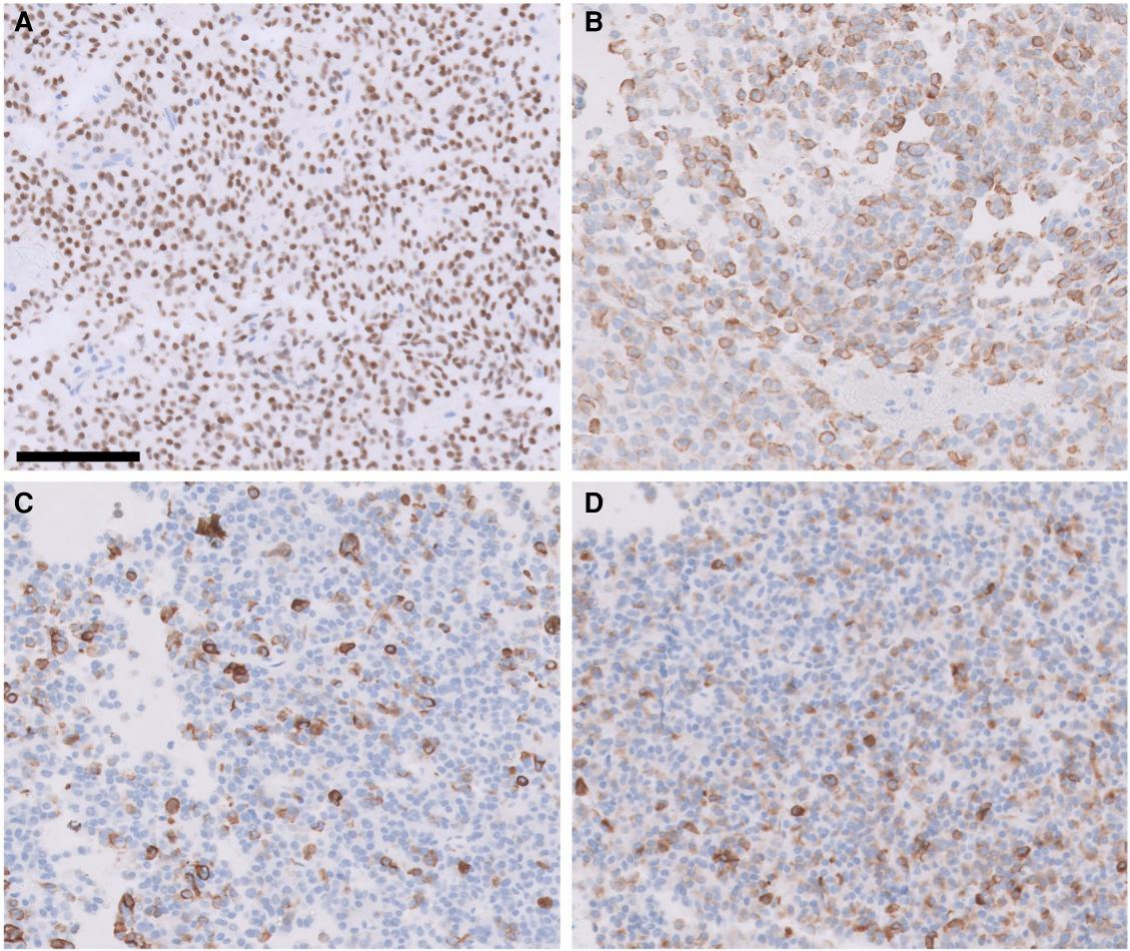
Immunohistochemistry consistent with mature plurihormonal pituitary-specific positive transcription factor 1–lineage pituitary adenoma; tumor cells are immunoreactive for pituitary-specific positive transcription factor 1 (A), GH (B), prolactin (C), and TSH (D). All images are taken at a total magnification of 200× (scale bar = 100 µm) and applies to all panels.

Pathology was revealing for a mature plurihormonal pituitary-specific positive transcription factor 1–lineage pituitary adenoma, with immunostaining of tumor tissue demonstrating positivity for pituitary-specific positive transcription factor 1, GH, TSH, and prolactin. It also stained negative for steroidogenic factor 1 (Fig. 2). The Ki-67 proliferation index was approximately 3% in tumor cells.

## Outcome and follow-up

A month after surgery, labs were obtained and were notable for a low TSH (0.16 mIU/L), normal free T4 (1.62 ng/dL [SI: 20.9 pmol/L]), downtrending IGF-1 (339 ng/mL [SI: 44.1 nmol/L]), and normal prolactin (15.1 ng/mL [SI: 320.1 mIU/L]). Given the TSH result, the patient was continued on levothyroxine 50 mcg once daily. Additionally, morning cortisol and ACTH levels remained normal (9.8 µg/dL [SI: 270.4 nmol/L]) and 44.2 pg/mL [SI: 9.7 pmol/L], respectively) after holding her morning dose of hydrocortisone.

She reported resolution of all presenting symptoms of thyrotoxicosis as well as a decrease in the size of her hands, feet, and nose. Her menstrual period returned spontaneously 3 months postoperatively.

The patient was lost to follow-up for 1 year and subsequently returned to clinic, having self-discontinued levothyroxine therapy. At 1-year follow-up, free T4 (1.57 ng/dL [SI: 20.2 pmol/L]) and TSH (1.120 mIU/L) were within normal limits, whereas IGF-1 remained persistently elevated (333 ng/mL, SI: 43.3 nmol/L). Given the persistent elevation in IGF-1, initiation of lanreotide 90 mg every 28 days was recommended. However, despite repeated follow-up and counseling, the patient declined treatment with lanreotide. At her most recent follow-up, 3 years postoperatively, IGF-1 had increased further (467 ng/mL [SI: 60.7 nmol/L]). Most recent pituitary MRI reveals a stable postoperative configuration of the sella with stalk deviated to the right into the residual pituitary tissue and hypoenhancing tissue in the left sella without evidence of new nodularity or recurrence.

## Discussion

This case demonstrates the potential for pituitary adenomas to secrete multiple biologically active pituitary hormones, including prolactin, TSH, and GH. It underscores the importance of maintaining a high index of suspicion for pituitary etiologies in the evaluation of thyrotoxicosis when TSH is not suppressed and of considering the plurihormonal potential of pituitary adenomas in patients presenting with multiple endocrinopathies. Prior studies indicate that up to 20% of TSH-secreting pituitary adenomas cosecrete GH [5, 6], whereas only 1.4% of GH-secreting pituitary adenomas cosecrete TSH [7]. In contrast, among clinically silent TSH-staining pituitary adenomas, plurihormonality is common, with as many as 60% costaining for GH, the most frequently associated hormone [8].

As observed in our patient, prior evidence suggests that plurihormonal pituitary adenomas present at a younger age than monohormonal tumors, highlighting the need for a comprehensive hormonal screen in younger patients with pituitary adenomas [7]. In 2 large Chinese cohorts, the age at diagnosis of mixed GH/TSH pituitary adenomas ranged from 19 to 59 years [7, 9]. Both studies demonstrated a male predominance among patients with plurihormonal tumors [7, 9]. The most common presenting symptoms reported include hyperthyroidism and acromegalic changes, such as altered facial appearance [7]. Thyroid eye disease is typically absent, whereas thyroid goiter is very common due to both TSH and IGF-1 stimulation of thyroid growth [9]. Biochemically, GH and TSH levels appear to be lower in plurihormonal pituitary adenomas compared with monohormonal tumors [7, 10]. However, in a more recent large Chinese study, IGF-1, FT3, and FT4 were not significantly different between plurihormonal and monohormonal pituitary adenomas [10]. Interestingly, our patient presented with amenorrhea and hyperprolactinemia. Although the degree of hyperprolactinemia is typically consistent with stalk effect, the tumor demonstrated positive prolactin staining on immunohistochemistry. Teramoto et al described a series of 20 TSH-secreting pituitary adenomas, in which prolactin levels were elevated in 3 patients, albeit at substantially lower levels than observed in our case (25-33 ng/mL [SI: 530-700 mIU/L] vs 135 ng/mL [SI: 2862 mIU/L]) [11]. Notably, none of the tumors with hyperprolactinemia in this series demonstrated concomitant GH hypersecretion. Similarly to our case, Morgante et al reported a case of a postmenopausal woman with a plurihormonal pituitary adenoma secreting GH, TSH, and prolactin with mildly elevated prolactin levels (43.5 ng/mL [SI: 922.2 mIU/L]) [12].

Surgical resection remains the first-line therapy for these tumors; however, previous studies have shown lower complete surgical remission rates and poorer overall prognosis [9, 13]. Compared with GH- or TSH-monohormonal tumors, plurihormonal pituitary adenomas tend to have a larger tumor diameter [7, 9]. It has been postulated that combined GH and TSH secretion may create permissive conditions for enhanced tumor growth [7]. Larger tumor size, more frequent cavernous sinus invasion, and increased intratumoral fibrosis collectively contribute to reduced rates of surgical remission [14]. A Ki-67 proliferative index greater than 3% has also been associated with worse prognosis for pituitary adenomas; notably, plurihormonal GH/TSH-secreting pituitary adenomas generally exhibit a lower Ki-67 index compared with GH-monosecreting tumors [10]. Nevertheless, among patients with mixed GH/TSH pituitary adenomas, a higher Ki-67 index was observed in those with incomplete remission [7]. Interestingly, in our patient, a Ki-67 index of 3% may suggest a need for closer and longer-term follow-up, especially given the persistence of IGF-1 elevation.

Both TSH- and GH-secreting pituitary adenomas express somatostatin receptors. Accordingly, in patients in whom surgery fails to restore euthyroidism and/or achieve normalization of GH or IGF-1 levels, adjunctive medical therapy with somatostatin analogs can be used. Indeed, in our patient, the persistent elevation of IGF-1 following surgery prompted consideration of somatostatin agonist therapy [6, 15]. A large Chinese study utilized a preoperative octreotide suppression test to assess hormonal responsiveness and potential therapeutic efficacy. Patients received a single 0.1 mg subcutaneous dose of octreotide, with TSH levels measured at 0, 2, 4, 6, 8, 24, 48, and 72 hours, and GH levels assessed at 0, 2, 4, 6, and 8 hours. Hormonal suppression was defined as a reduction of both GH and TSH to less than 50% of baseline values. Using these criteria, octreotide reduced GH and TSH levels by 79.1% and 94.7%, respectively [7]. Additionally, several studies proposed the use of octreotide as the initial treatment to achieve biochemical hormonal control [7, 9]. Specifically in thyrotroph pituitary adenomas, somatostatin analogs have been used to improve hormone levels preoperatively and prevent thyroid storm, improve visual field defects, and reduce tumor size [9, 16]. High cost is a frequent barrier to long-term use, reinforcing surgical resection as the preferred first-line therapy. In patients who do not achieve a cure with or who have contraindications to surgical/medical therapy, stereotactic radiotherapy has been used [15, 17]. However, its effectiveness for patients with plurihormonal (GH and TSH) pituitary adenomas is uncertain, and its use is often associated with high incidences of hypopituitarism [9, 10].

Data on long-term recurrence rates among patients who are determined to be cured following surgery are limited, although recurrence appears to be uncommon in the first years after surgery [13]. Interestingly, data have shown that some patients will achieve remission in 1 of the 2 hormonal axes, but not the other, as observed in our patient [9]. Accordingly, patients considered cured should have regular, ongoing monitoring for evidence of clinical, biochemical, or radiologic recurrence. Prior studies have demonstrated that IGF-1 levels may remain elevated for months following surgery; therefore, assessment of GH levels after a 75-g glucose load may be more informative in short-term assessment [9, 18].

In conclusion, plurihormonal pituitary adenomas cosecreting GH, prolactin, and TSH are rare tumors with heterogeneous clinical presentations and more aggressive behavior compared with monohormonal pituitary adenomas, particularly when associated with a relatively high Ki-67 index. Optimal management requires a high index of suspicion, comprehensive hormonal evaluation, and a multimodal treatment approach centered on surgical resection, with adjunctive medical and radiotherapeutic therapies as needed. Given the potential for incomplete remission and delayed recurrence, long-term clinical, biochemical, and radiologic surveillance remains essential.

## Learning points

- TSH- and GH-secreting pituitary adenomas are both rare entities, and tumors that cosecrete biologically active TSH, prolactin, and GH are even rarer.
- Consider the plurihormonal potential of pituitary adenomas in patients presenting with multiple endocrinopathies
- Transsphenoidal surgery remains the first-line treatment for cosecreting pituitary adenomas

## Contributors

All authors made individual contributions to authorship. D.S., A.C.L., and R.S. were involved in the diagnosis and management of the patient. T.E.R. and R.T.Y. were involved in histopathology section and preparation of histology images. R.S. was responsible for the patient's surgeries. J.F.C was responsible for synthesis of clinical findings and literature review. J.F.C. and D.S. drafted the manuscript. All authors reviewed and approved the final draft.

## Data Availability

Data sharing is not applicable to this article as no datasets were generated or analyzed during the current study.
